# Function and prognostic value of basement membrane -related genes in lung adenocarcinoma

**DOI:** 10.3389/fphar.2023.1185380

**Published:** 2023-05-05

**Authors:** Yurong Zhang, Tingting Li, Huanqing Liu, Li Wang

**Affiliations:** ^1^ Department of Scientific Research, The First Affiliated Hospital, Xi’an Medical University, Xi’an, Shaanxi, China; ^2^ Department of Pharmacy, Xi’an Chest Hospital, Xi’an, Shaanxi, China; ^3^ Information Construction and Management Office, Northwest Polytechnical University, Xi’an, Shaanxi, China

**Keywords:** basement membrane, LUAD, prognosis, potential drug candidate, risk score

## Abstract

**Background:** Lung adenocarcinoma (LUAD) has become a common cause of cancer-related death. Many studies have shown that the basement membrane (BM) is associated with the development of cancer. However, BM-related gene expression and its relationship to LUAD prognosis remains unclear.

**Methods:** BM-related genes from previous studies were used. Clinical and mRNA expression information were obtained from TCGA database. Cox, minimum absolute contraction, and selection operator regression were applied to analyze the selected genes affecting LUAD prognosis. A prognostic-risk model was then established. Furthermore, this study applied Kaplan-Meier analysis to assess the outcomes of high- and low-risk groups, then explored their differences in drug sensitivity. The DSigDB database was used to screen for therapeutic small-molecule drugs.

**Results:** Fourteen prognostic models based on BM-related genes were successfully constructed and validated in patients with LUAD. We also found that independence was a prognostic factor in all 14 BM-based models. Functional analysis showed that the enrichment of BM-related genes mainly originated from signaling pathways related to cancer. The BM-based model also suggested that immune cell infiltration is associated with checkpoints. The low-risk patients may benefit from cyclopamine and docetaxel treatments.

**Conclusion:** This study identified a reliable biomarker to predict survival in patients with LUAD and offered new insights into the function of BM-related genes in LUAD.

## 1 Introduction

Served as a common cancer, lung cancer (LC) has become one of the leading causes in humans, with an estimated 5-year survival rate of 19% (Siegel et al., 2020). Non-small cell lung cancer (NSCLC) accounts for about 90% of the total number of LC cases, of which lung adenocarcinoma (LUAD) is considered to be one of the most common histological subtypes of LC. According to statistics, the incidence of LUAD is increasing year by year. Although the prognosis of LUAD continues to improve with the advancement of targeted molecular therapy and immunotherapy, the improvement in LUAD therapeutic outcomes is limited to some patients with specific molecular characteristics, such as driver gene mutations in EGFR (Herbst et al., 2018; Duma et al., 2019). Therefore, an accurate and individualized assessment and the improvement of survival in patients with LUAD remain major challenges.

The basement membrane (BM) plays a crucial role in normal tissues (Yurchenco, 2011), and its proteins participate in the formation, invasion, as well as metastasis of cancer cells. Relevant reports have proposed that stromal cell invasion is essential for the division and growth of tumor cells. BM-related genes have a significant influence on the invasiveness of cancer cells in the oral cavity (Celentano et al., 2021), breast (Liu et al., 2018), ovary (Januchowski et al., 2016), etc. Serving as one of the most destructive types of malignancy, the progression of lung cancer is strongly related to the interaction between cells and their matrix environments. Matric stiffness associated with enhanced collagen crosslinking may affect the motility of lung cancer cells (Götte and Kovalszky, 2018). XIX collagen, with certain relevance to types XVIII and XV in the BM zone, has been shown to enhance several types of cancer, distinguishing NSCLC from healthy controls (Liu et al., 2016). XVII collagen has been shown to be essential for maintaining epithelial-Mesenchymal transition (EMT) phenotypes and metastasis in lung cancer stem-like cells. Col XVII inhibits ubiquitination degradation in snails by activating the FAK/AKT/GSK3β pathway by stabilizing laminin-5. Col XVII and Laminin-5 were overexpressed in patients with LC resection, and the prognosis was the worst (Liu et al., 2016). Laminin, the main structural component of BM, is a strong promoter that promotes cell adhesion, migration, differentiation and proliferation through integrins and other cell receptors on the surface. Studies have supported the importance of serum laminin levels as a diagnostic marker in LC patients. Laminin ɣ2 is different melamine adhesion protein 332 a unique subunit, the level in patients with NSCLC was found to be enhanced. Laminin ɣ2 with NSCLC was significantly associated with poor prognosis, especially for the early cases (Tas et al., 2016; Teng et al., 2016). Therefore, the speculation that BM-related genes may be closely related to the occurrence and development of LUAD is worth further study, and these proteins have been identified as biomarkers that can be used to predict, diagnose, or treat LUAD.

In recent decades, great strides have been made in the field of BM, from understanding basic BM-related cancer characteristics to identify the underlying genetic changes that drive unique gene expression programs and promote tumor development. Therefore, it is necessary to better understand the effects of BM on the progression and treatment of LUAD to lay the foundation for further research. However, few studies mentioned the relationship between BM related genes and progression of LUAD. This study investigated changes in BM-related gene expression in both LUAD and normal lung tissues and their prognostic value for LUAD based on bioinformatics methods. Using statistical methods, a prognostic feature was successfully constructed and verified based on 14 BM-related genes, which may predict the prognosis of LUAD in an effective way. Additionally, we also demonstrated the prognostic characteristics of LUAD were correlated with the immune microenvironment, offering a theoretical basis for the application of immune checkpoint in LUAD therapy. Subsequently, Twelve small molecule drugs that may participate in LUAD therapy were found, opening a new path for the development of drugs to treat LUAD.

## 2 Materials and methods

### 2.1 Acquire data and patient samples

The study acquired clinicopathological data and mRNA expression of LUAD patients from TCGA database (https://cancergenome.nih.gov/), namely, sex, age, staging status, TMN type, survival status, and survival duration. Finally, the relevant information of normal lung tissues of 19 patients and LUAD tissues of 414 patients were screened from the TCGA database. Total of 224 BM-related genes were identified through a literature search (Jayadev et al., 2022).

### 2.2 Identify differentially expressed BM

The R package was applied for the normalization of the collected mRNA expression profiles. And BM-related differentially expressed genes were screened using Limma package of R software within the scope of | logFC | > 1 and FDR < 0.05.

### 2.3 Functional enrichment analyses

ClusterProfiler software packages were utilized for performing gene ontology (GO) analyses, including Cell Composition (CC), Molecular Functionality (MF), and Biological Process (BP). Kyoto Encyclopedia of Genes and Genomes (KEGG) pathway analyses were carried out using the same software. These were regarded as significantly enriched (FDR and *p* < 0.05).

### 2.4 Establish PPI network

This study submitted varying expressed BM-related genes to the Search Tool for the Retrieval of Distant Genes Database (STRING) (https://cn.string-db.org/) to obtain information about protein-protein interaction, and visualized the PPI network through Cytoscape software (Ver. 3.7.1). Molecular Complex Detection (MCODE) (Ver. 1.6.1) is a Cytoscape plug-in that identifies regions with dense connections and selects statistically significant models. The key modules with MCODE were identified, of which MCODE score>5, degree cut-off =2, node cut-off = 0.2, maximum depth = 100, and K-score =2.

### 2.5 Screen out potential small molecule medication

The differentially expressed genes were updated to the Connectivity MAP database (CMAP) (https://portals.broadinstitute.org/cmap/) to screen out potential BM-related small molecule drugs, which may be used for treating patients with LUAD. The proximity (−1<p< 1) of compounds associated with the uploaded genes was evaluated accordingly. Thus, it can be concluded that negative drugs can inhibit cancer cells. The threshold values were as follows: *p* < 0.01, N ≥ 3%, non-null =100, and enrichment < −0.8.

### 2.6 Develop and verify the prognosis model based on BM

The prognostic value of BM-related genes was preliminarily assessed using univariate COX regression. Multivariate COX proportional hazards regression model was applied to access those obtained data. The prognostic value of BM-related genes was first assessed by univariate COX regression. Genes obtained from univariate COX regression model and clinical factors were used in multivariate COX proportional hazard regression model. Only BM genes and clinical factors in the univariate and multivariate COX analyses with *p* < 0.05 were deemed as prognosis factors of LUAD. Then LASSO analysis was employed for constructing prognostic risk model via the glmnet R package. Calculation of risk scores was done through utilization tools with the formula as follows:

$$\begin{array}{ll} Risk\;score & =\left(coefficient\;mRNA1\times expression\;of\;mRNA1\right)\\ & +\left(coefficient\;mRNA2\times expression\;of\;mRNA2\right)\\ & +\cdots +\left(coefficient\;mRNAn\times expression\;mRNAn\right) \end{array}$$

Patients with LUAD were divided into two groups (high- and low-risk) according to their median risk score. This study used Kaplan-Meier analysis to analyze and compare OS times and applied the surviving ROC package to plot time-dependent receiver operating characteristic (ROC) curves and predict the accuracy of prognostic indicators. To verify the prognostic value of BM-based characteristics, we randomly selected 30% of TCGA sample data for validation using the same method.

### 2.7 Conduct gene set enrichment analysis

Gene Set Enrichment Analysis (GSEA) has been applied for exploring potential molecular mechanisms in low- and high-risk populations. Those with *p* < 0.05 and FDR < 25% should be thought to be differences with statistical significance.

### 2.8 Nomogram construction

The relationship between signatures based on BM-related genes and clinical features was investigated. The univariate and multivariate COX regression analyses were conducted to investigate whether risk scores had independent prognostic value among with LUAD suffers. Clinical characteristics with BM-based signature risk scores were deployed to establish nomogram prognostic maps for LUAD patients with OS of 3 and 5 years.

### 2.9 Immune cell infiltration analysis

Many algorithms, including McP-counter, ciber-SORT, Cibersor-ABS, QUANTISEQ, XCELL, etc. were applied to assess immune cell infiltration levels in the two groups. To predict the efficacy of immune checkpoints in blocking therapy, we examined the expression of immune checkpoints, such as BTLA, BTNL2, CD27, CD28, CD40LG, and TNFSF15. In addition, the connection between immune cells and 14 BM-related genes was identified through TIMER database (https://cistrome.shinyapps.io/timer/), improving our acquaintance of the functionality of BM-related genes in LUAD.

### 2.10 Medication sensitivity analyses

The Genomics of Drug Sensitivity in Cancer (GDSC) database (http://www.Cancerxgene.org/) was applied to investigate differences in drug sensitivity, then analyzed the half-maximal inhibitory concentration (IC50) of medication, aiming to predict its sensitivity through the package (pRRophetic). Those with *p* < 0.05 had statistical significance.

### 2.11 Statistical analyses

Univariate analysis of variance was used to compare gene expression levels in normal lung tissue and LUAD tissue, and Pearson Chi-square test was used to compare categorical variables. To compare OS in different subgroups, Kaplan-Meier method and two-sided log-rank test were used. To assess the independent prognostic value of the risk model, univariate and multivariate Cox regression models were used. Through using Wilcoxon test, distinctions between the two groups were contrasted. *p* < 0.05 meant statistical significance. The Mann-Whitney test was used to compare the immune cell infiltration and immune pathway activation between the two groups. The statistical analysis was performed by using R software (Ver.4.0.5).

## 3 Results

### 3.1 Establish and validate BM-based signature

**FIGURE 1:**
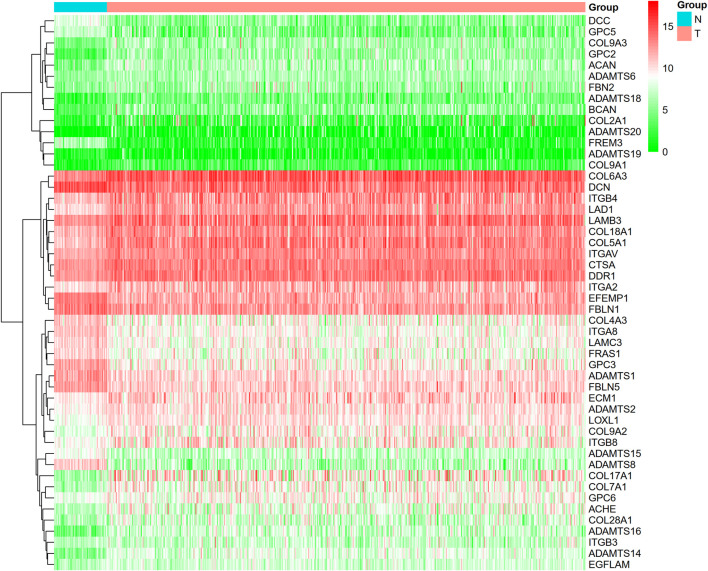
Heatmap revealed BM with differential expressions. (green color: low expression; red color: high expression).

**FIGURE 2:**
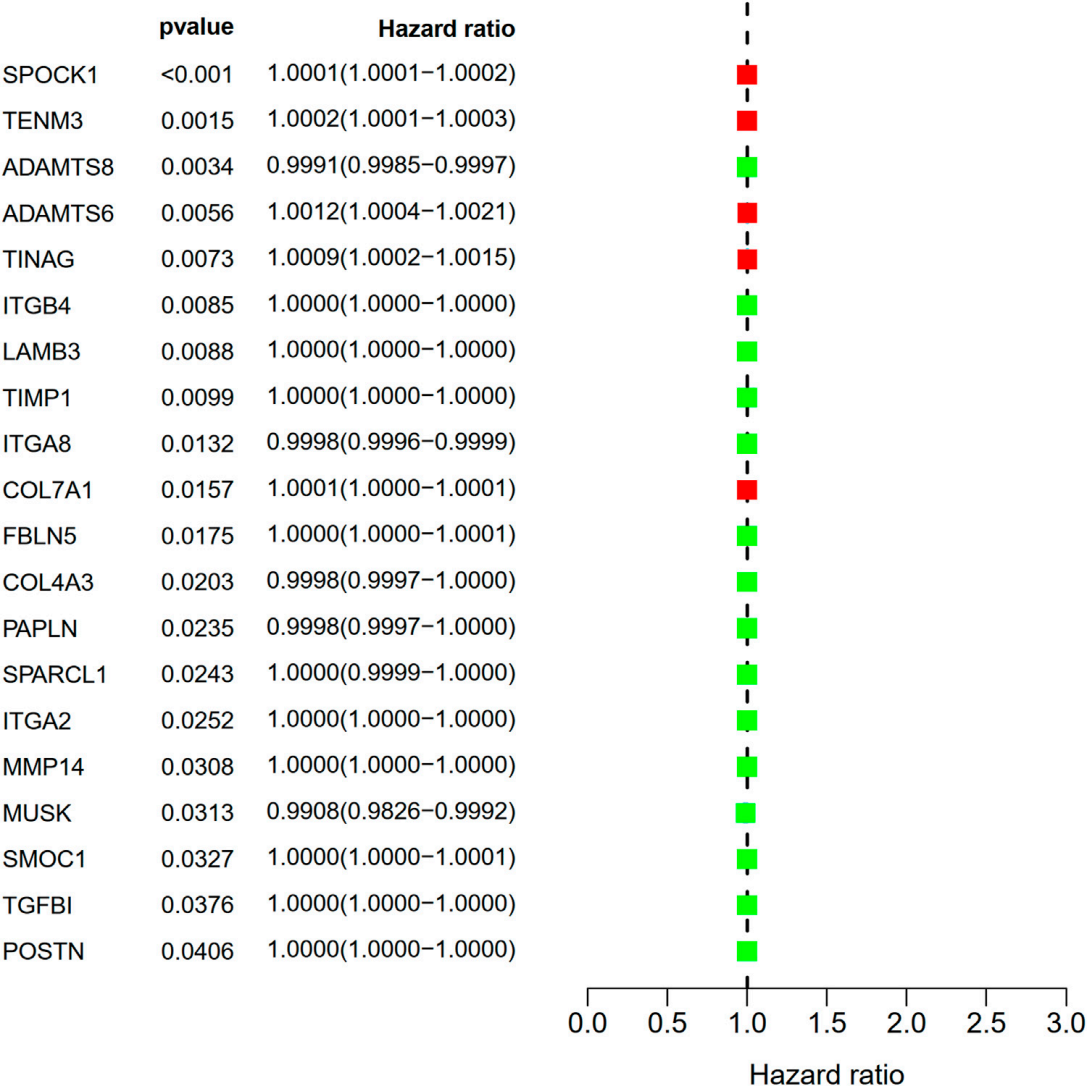
Identification of prognostic BM related genes by univariate Cox regression analysis.

**FIGURE 3:**
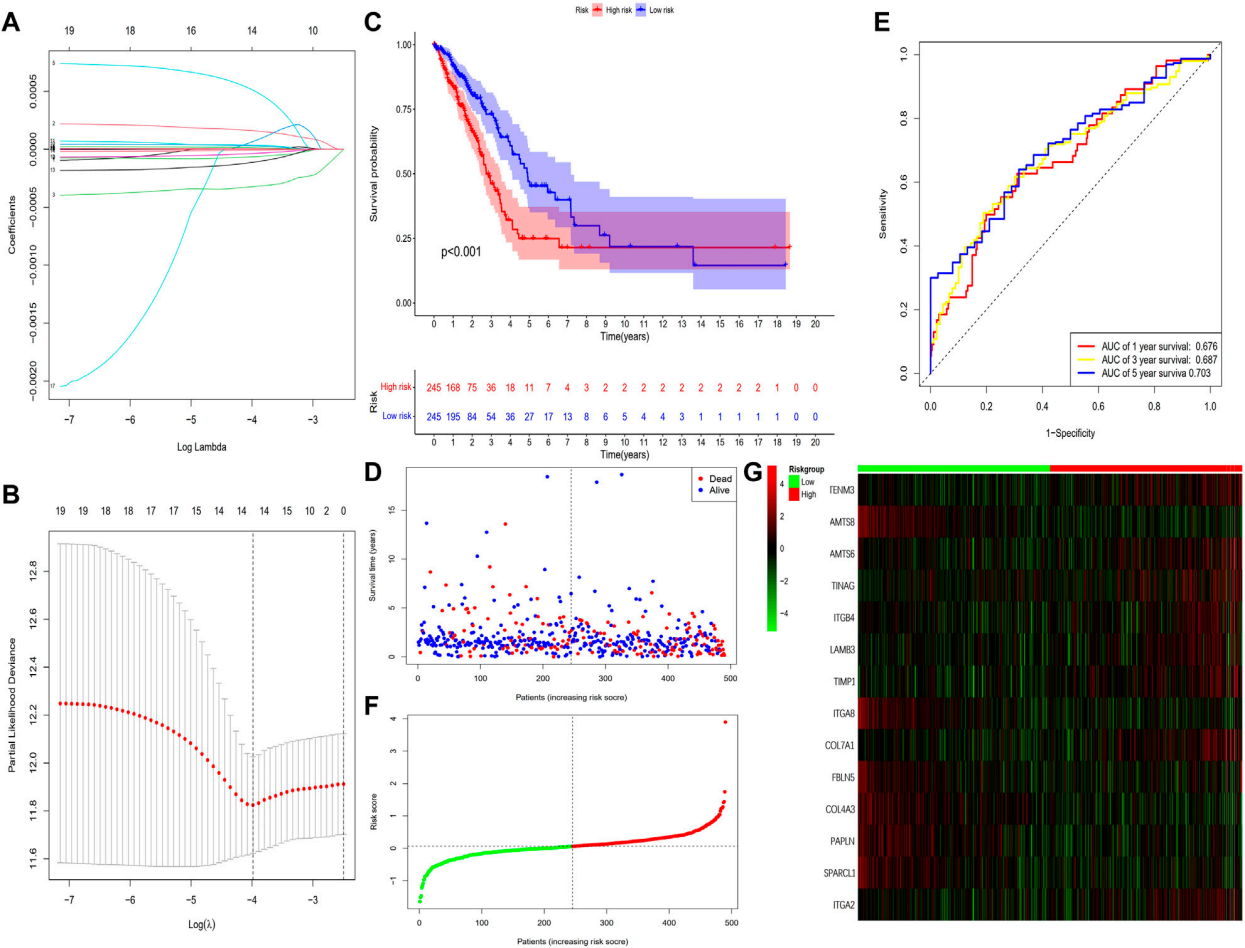
Construction of the prognostic BM-based signature in TCGA cohort. **(A)** Identification of prognostic BM related genes by univariate Cox regression analyses. **(B)** LASSO regression of the 14 BM-related genes. **(C)** Cross-validation of the parameter selection adjustment in the LASSO regression. **(D)** Kaplan-Meier survival analysis of LUAD patients between high-risk groups and low-risk groups. **(E)** Distribution of survival status based on the median risk score. **(F)** ROC analysis of risk scores predicting the OS. **(G)** Heatmap showed the differences of 14 BM related genes between high and low-risk patients.

This study identified 93 differentially expressed BM-related genes, of which 31 were downregulated BM related genes and 62 were upregulated in the TCGA-LUAD dataset. The expression of the identified BM related genes is presented as heat maps in Figure 1. According to the abnormal expression value of BM related genes obtained, univariate COX regression analysis was used to probe the prognostic value of BM related genes in LUAD. The results suggested that only 20 genes had prognostic value (Figure 2). The LASSO COX regression analysis was carried out to establish the prognostic significance in LUAD patients. It should be mentioned that a risk model was constructed with a total of 14 genes (TENM3, ADAMTS8, ADAMTS6, TINAG, ITGB4, LAMB3, TIMP1, ITGA8, COL7A1, FBLN5, COL4A3, PAPLN, SPARCL1, and ITGA2) (Figures 3A,B). Calculation of the risk score was conducted using the corresponding coefficients of the 14 BM genes according to the following formula (see Table 1):

$$\begin{array}{ll} Risk\;Score & =\left(1.60E-04\times TENM3\;expression\right)+\left(-3.23E-04\times ADAMTS8\;expression\right)\\ & +\left(6.64E-05\times ADAMTS6\;expression\right)+\left(1.30E-04\times TINAG\;expression\right)\\ & +\left(2.46E-06\times ITGB4\;expression\right)+\left(1.49E-06\times LAMB3\;expression\right)\\ & +\left(4.70E-05\times TIMP1\;expression\right)+\left(-6.51E-05\times ITGA8\;expression\right)\\ & +\left(2.9E-06\times COL7A1\;expression\right)+\left(3.54E-05\times FBLN5\;expression\right)\\ & +(-3.63E-05\times COL4A3\;expression+(-1.12E-04\times PAPLN\;expression\\ & +(-8.54E-06\times SPARCL1\;expression+\left(1.39E-05\times ITGA2\;expression\right) \end{array}$$

**TABLE 1:** Gene list and coefficient.

Gene symbol	Coefficient
TENM3	1.60E-04
ADAMTS8	−3.23E-04
ADAMTS6	6.64E-05
TINAG	5.10E-04
ITGB4	2.46E-06
LAMB3	1.49E-06
TIMP1	4.70E-06
ITGA8	−6.51E-05
COL7A1	2.90E-05
FBLN5	3.54E-05
COL4A3	−3.63E-05
PAPLN	−1.1E-04
SPARCL1	−8.54E-06
ITGA2	1.39E-05

**FIGURE 4:**
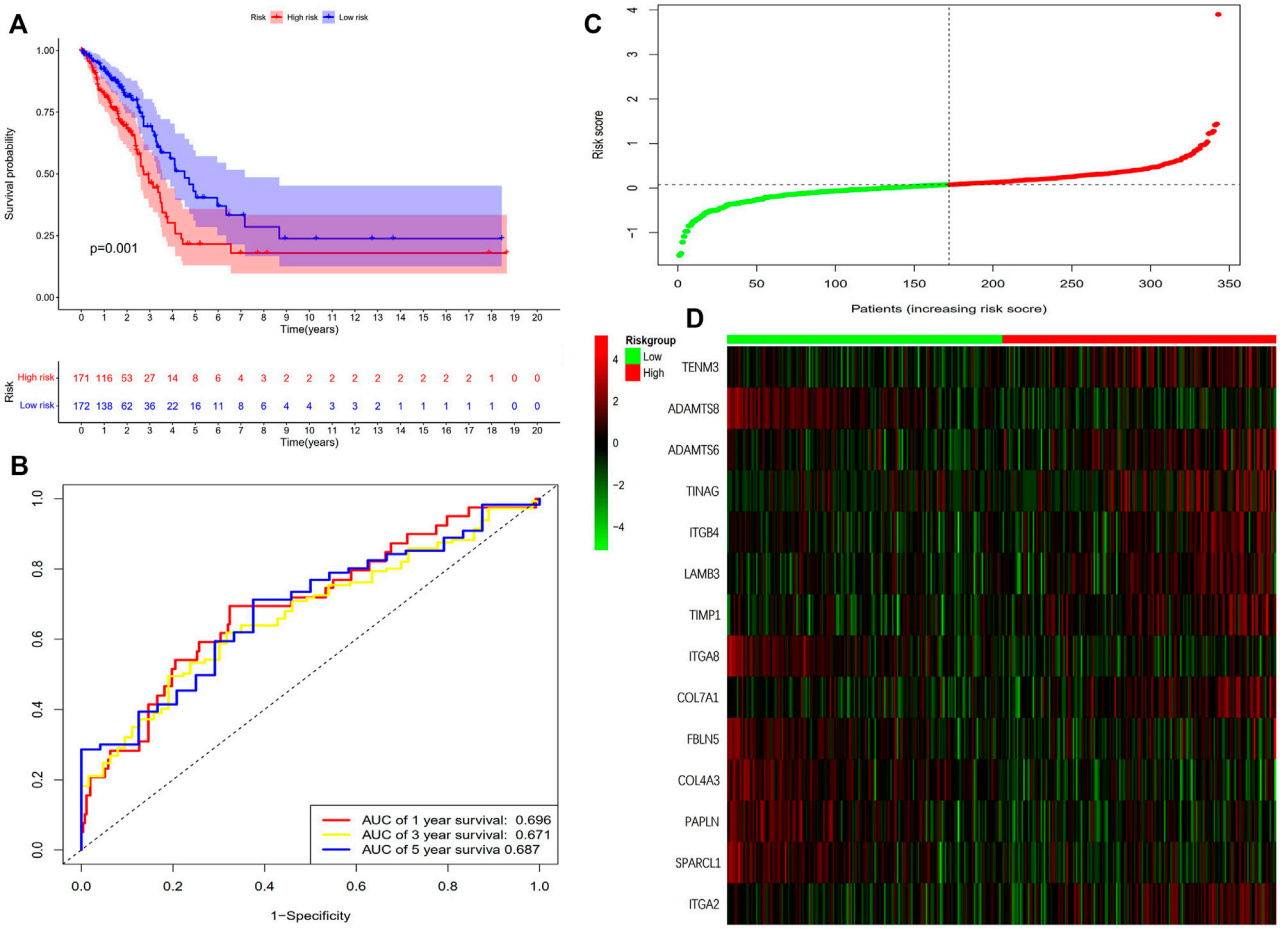
Validation of the prognostic BM-based signature. **(A)** Kaplan-Meier survival analysis of LUAD patients between high-risk groups and low-risk groups; **(B)** Time-independent receiver operating characteristic (ROC) analysis of risk scores predicting the overall survival; **(C)** Distribution of survival status based on the median risk score; **(D)** Heatmap showed the differences of 14 BM related genes between high and low-risk patients.

The results showed that the mortality rate of high-risk patients was significantly higher than that of low-risk patients (*p* < 0.001), suggesting risk scores showed a negative correlation with prognosis (Figures 3C–E). The study used time-dependent ROC analysis to evaluate the specific with sensitive features in prognostic model. And AUCs were 0.676, 0.687, and 0.703 for 1-year, 3-year, and 5-year survival, respectively (Figure 3F). Additionally, we plotted a heatmap of the 14 BM-related genes in patients in the high-risk and low-risk TCGA groups (Figure 3G). To verify the prognostic value of BM-based characteristics, we randomly selected 30% of TCGA sample data for validation using the same method (Figure 4).

### 3.2 Independent prognosis values of the risk model

**FIGURE 5:**
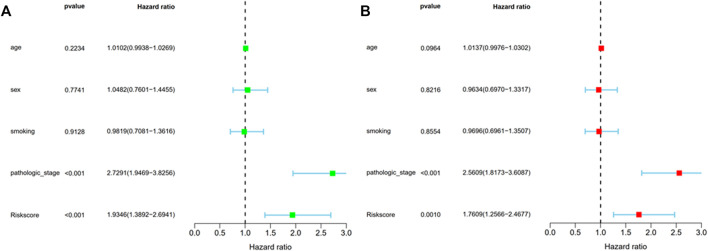
Prognosis factor with independency for LUAD in the TCGA cohort. **(A)** The correlations of the risk score for OS with clinical pathology factors through Univariate Cox regression analyses; **(B)** The correlations of the risk score for OS with clinical pathology factors through Multivariate Cox regression analyses.

The study carried out univariate and multivariate COX analyses to screen out signatures that may be used as independent prognostic indicators. The former indicated that two factors, including risk score and pathologic stage showed a significant correlation with the survival of patients with LUAD (*p* < 0.001) (Figure 5A). The latter showed that these two factors were closely associated with prognosis (*p* < 0.05) (Figure 5B). The above mentioned results suggested BM-based signatures are independent prognostic indicators in patients with LUAD.

### 3.3 Relationship between clinical characteristics and signatures

**FIGURE 6:**
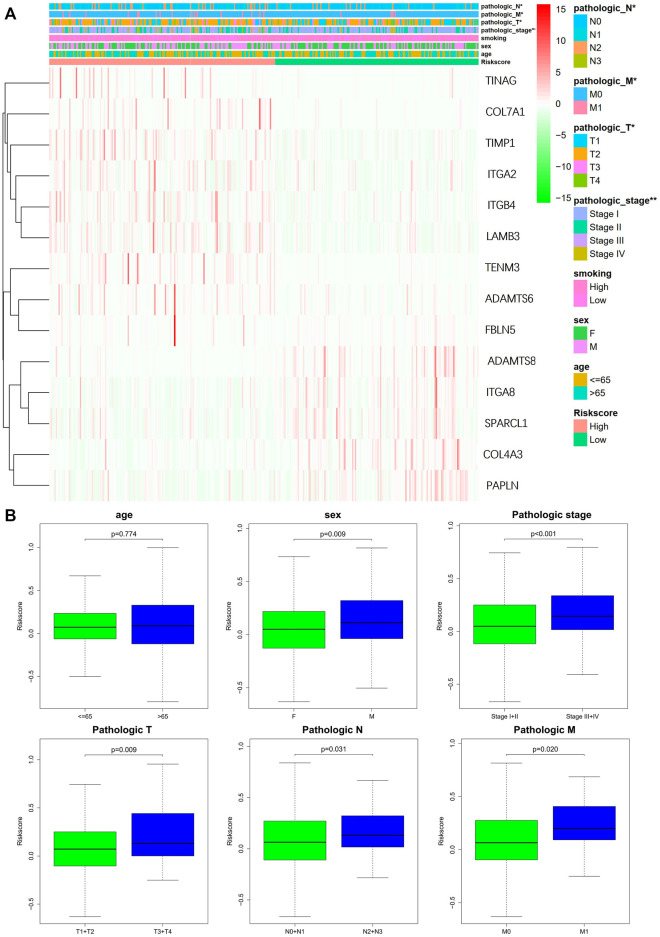
Correlation of signature with clinical features. **(A)** Chi-square test of LUAD patients between high- and low-risk groups. **(B)** Stratified analysis of clinical subgroups: prognostic significance of age, sex, and TNM stage.

**FIGURE 7:**
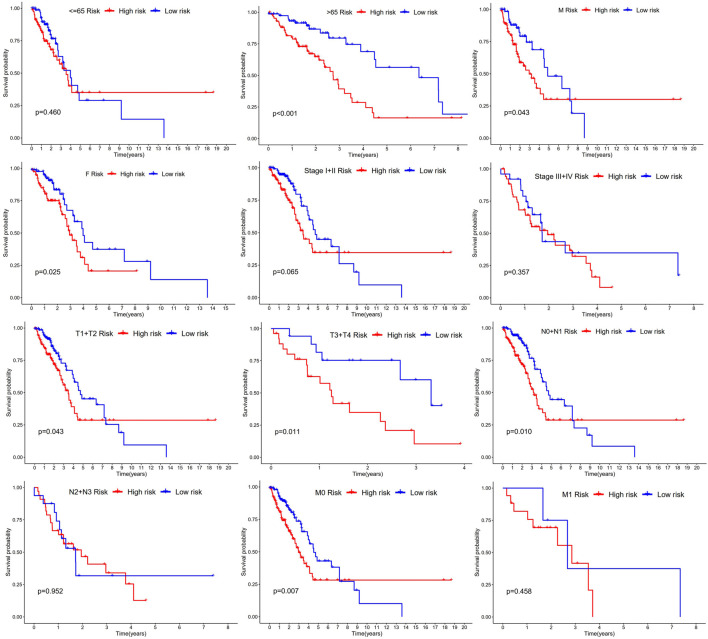
Kaplan-Meier curves of OS differences through stratification of age, gender, grade, and TNM stage between the high-risk groups and low-risk groups.

The study used Chi-square test to probe into whether the prognostic signature was involved in LUAD progression and development. According to the results, sex (*p* < 0.05), pathologic stage (*p* < 0.001), T stage (*p* < 0.05), M stage (*p* < 0.05), and N stage (*p* < 0.005) were statistically significant, whereas there was no difference statistical in age (*p* > 0.05) (Figures 6A,B). Furthermore, this study conducted a stratified analysis to explore its prognostic significance in some subgroups, such as age, sex, and TNM stage. The results showed that BM-based signatures performed well in those with age>65 years (*p* < 0.001), with female *p* = 0.025 and male *p* = 0.043, together with T1–T2 *p* = 0.043, T3–T4 *p* = 0.011, N0–N1 *p* = 0.01, and M0 *p* < 0.001. However, the BM-based signature had poor predictive ability in age ≤ 65 years (*p* = 0.460), stages I–II (*p* = 0.062), stages III–IV (*p* = 0.357), and N2–N3 (*p* = 0.0.952) (Figure 7).

### 3.4 Establish a nomogram

**FIGURE 8:**
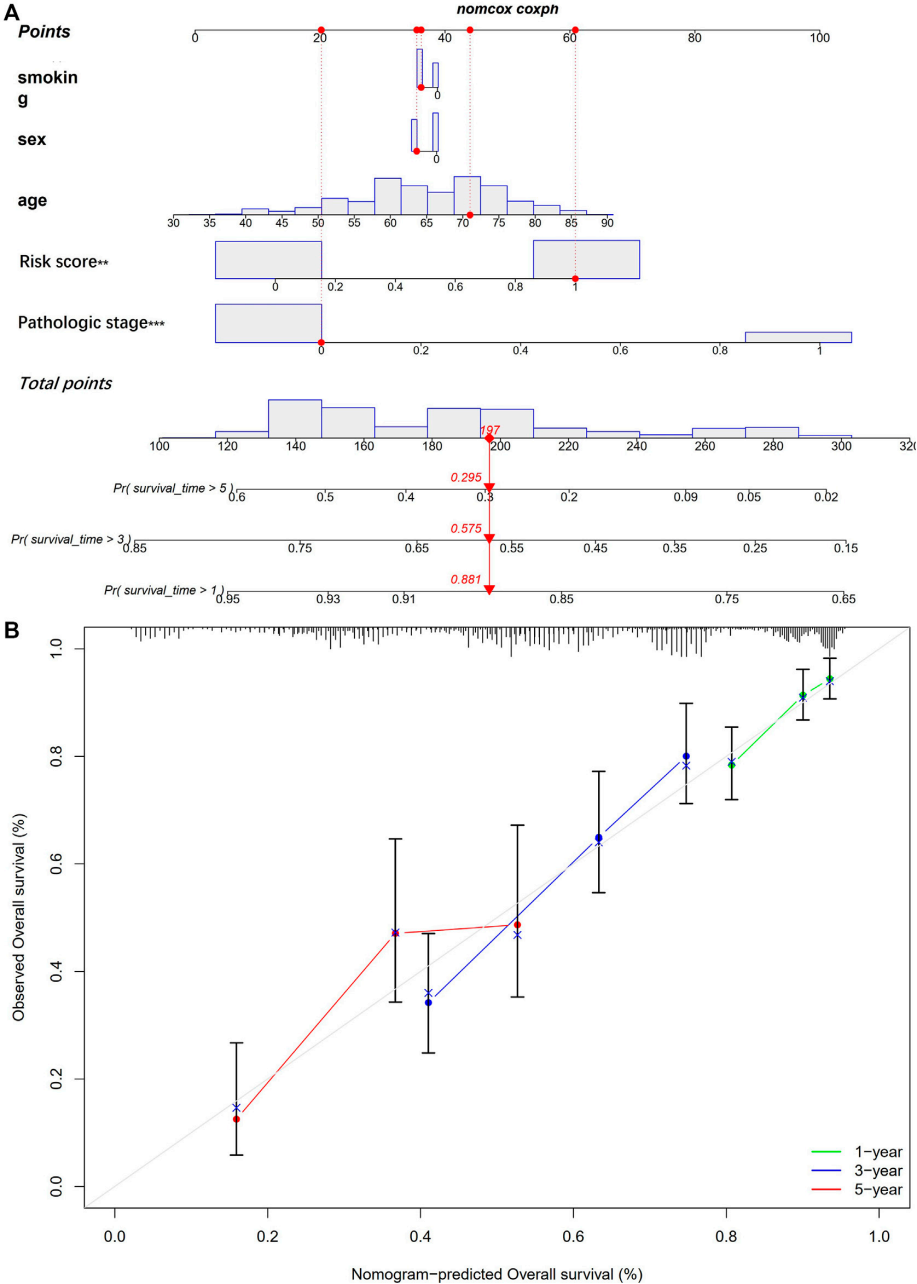
Establishment of a nomogram. **(A)** nomogram for prediction of 1-, 3- and 5-year OS; **(B)** The calibration plots for prediction of 1-, 3- and 5-year OS.

The nomogram includes multiple prognostic indicators and can be used to graphically assess individual survival likelihood, which aimed to predict the survival of patients with LUAD. Its indicators included sex, smoking status, age, pathological stage, and risk score (Figure 8A). The results indicated that actual survival was fitted with the predicted value (Figure 8B).

### 3.5 Functional enrichment analysis and protein-protein interaction (PPI)

**FIGURE 9:**
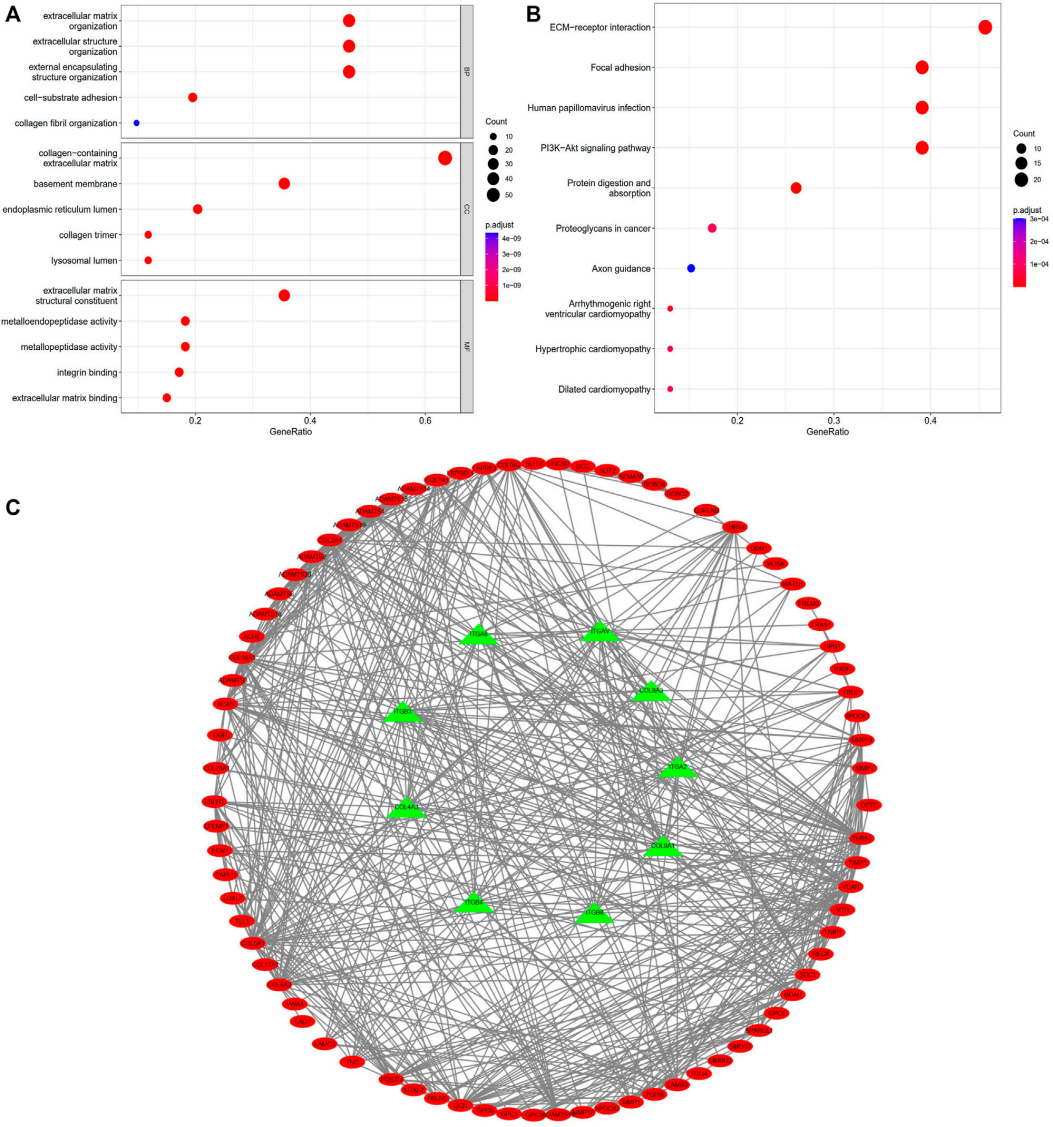
Enrichment analyses of differentially expressed BM related genes. **(A)** GO analysis; **(B)** KEGG analysis. **(C)** PPI network of BM related genes with differential expression.

GO is a bioinformatics tool for the annotation of functional studies, which is widely applied in Molecular Function (MF), Biological Process (BP), and Cellular Components (CC), whereas KEGG is a repository of genetic network information generated by high-throughput experimental techniques. The potential functions of BM-related genes with different expression levels among the subgroups of the risk model classification were explored using GO and KEGG analyses. We identified 93 BM genes from the two groups in TCGA cohort. BP analysis showed these genes remarkably participated in an extracellular matrix organization and structure organization as well as external encapsulating structure organization; According to CC analyses, collagen with the extracellular matrix, basement membrane and endoplasmic reticulum lumen was commonly enriched; the result of MF analyses showed 93 BM-related genes were primly related to extracellular matrix structural constituent, metalloendopeptidase activity, and metallopeptidase activity (Figure 9A). According to KEGG pathway analysis, ECM−receptor interaction, PI3K−Akt signaling pathway were mainly involved (Figure 9B). The STRING database indicated that the BM PPI network with differential expression consisted of 93 nodes and 582 edges (Figure 9C).

### 3.6 GSEA analysis

**FIGURE 10:**
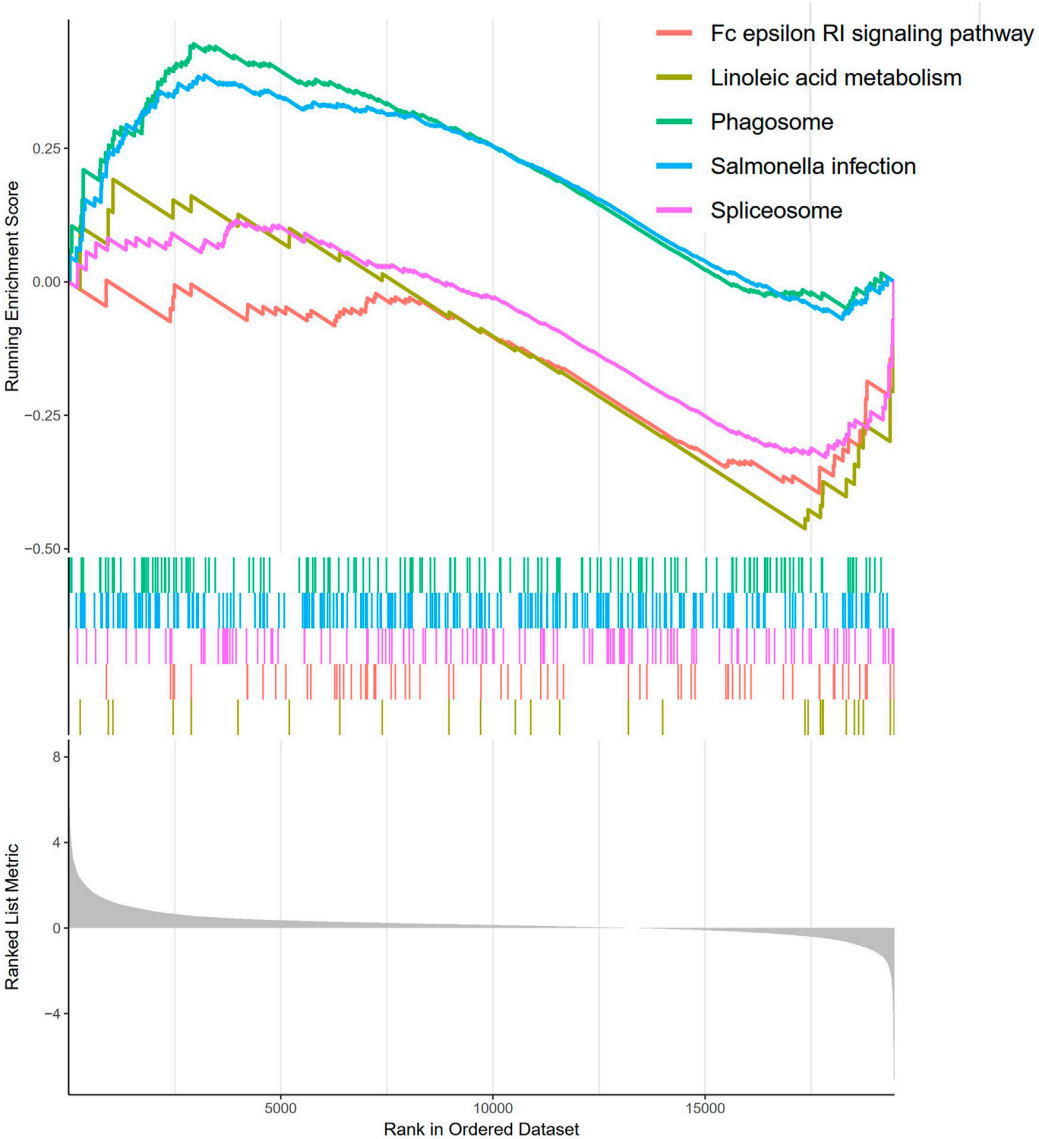
GSEA analysis of BM-based signature.

The molecular mechanism of BM-based signature was clarified based on GSEA analysis. The results indicated that the Fc epsilon RI signaling pathway, linoleic acid metabolism, phagosomes, *Salmonella* infection, and spliceosome got main enrichment in high-risk group (Figure 10).

### 3.7 Immune infiltration level analysis on BM-based signature

**FIGURE 11:**
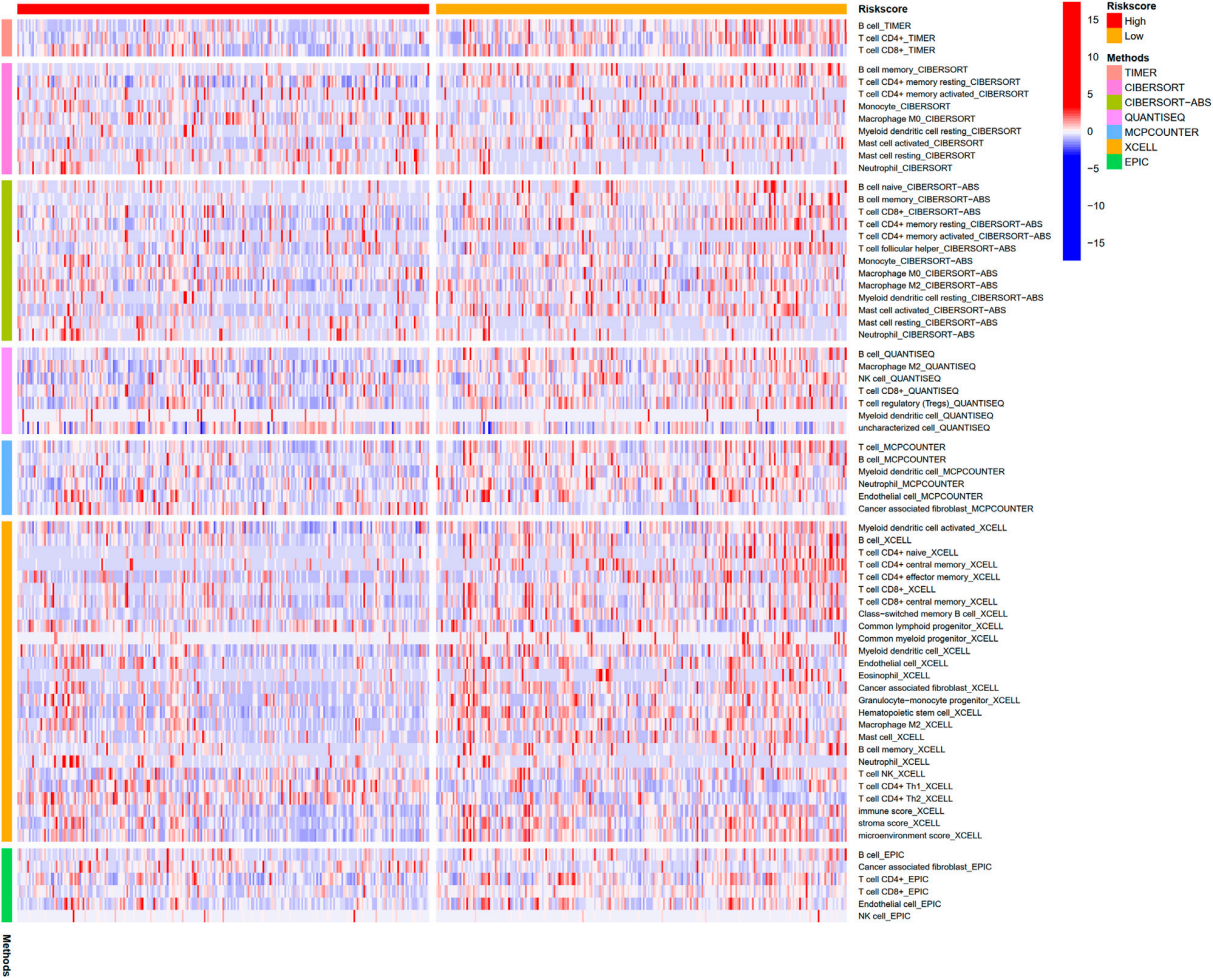
Immunity cells infiltration amid high-risk groups and low-risk groups.

**FIGURE 12:**
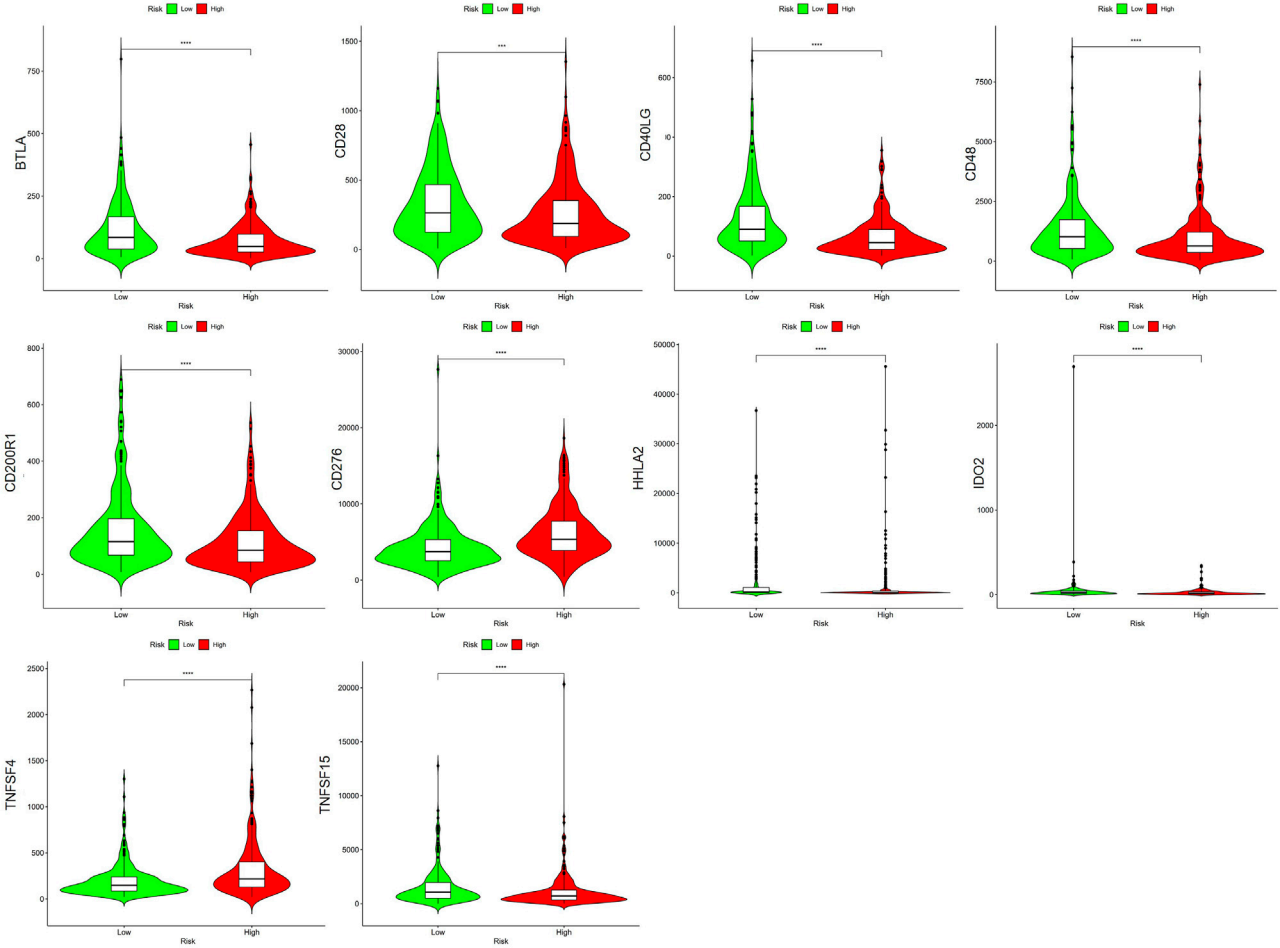
The relationship of prognosis feature with immunity checkpoints.

We explored the connection between BM-based signature and immunity infiltration based on these following analyses; namely, CIBERSORT, TIMER, CIBERSORT, CIBERSORT-ABS, XCELL, EPIC, and MCP-counter analyses, and the outcomes were demonstrated in the heatmap (Figure 11). According to the results of CIBERSORT, the low-risk group patients had higher proportions of B cells, CD4+ T cells, monocytes, myeloid dendritic cells, and mast cells, whereas the high-risk group patients had higher ratios of M0 macrophages and neutrophils. Considering the importance of checkpoint inhibitor immunotherapy, this study carried out the analysis on the relation between key immune checkpoints and risk score (Danilova et al., 2019). More importantly, there exited a great difference in the expression of BTLA, CD28, CD40LG, CD48, CD200R1, CD276, HHLA2, IDO2, TNFSF4, and TNFSF15 between patients in the two groups. Additionally, immune checkpoints were all expressed in high-risk patients, indicating immunosuppressive and failure phenotypes (Figure 12).

### 3.8 Identify small molecule drugs

This study acquired 12 potential small molecule drugs based on BM from the DsigDB database, including 1 h-pyrazolo[3,4-d] pyrimidine, progesterone, phenytoin, 8-Bromo-cAMP Na, Indeno[1,2,3-cd] pyrene, LAMININ BOSS, LY-294002 PC3 UP, aspirin, Aflodac, anthracene, lamivudine and fluoranthene (Table 2).

**TABLE 2:** The 12 small molecular medication of DsigDB database analysing outcomes.

Term	*p*-value	Odds ratio	Combined score
1 h-pyrazolo[3,4-d]pyrimidine	9.59E-06	93.70532915	1082.71
progesterone	1.23E-05	12.64044747	142.89
phenytoin	5.49E-05	51.14922813	501.81
8-Bromo-cAMP Na	5.68E-05	16.60570153	162.33
Indeno[1,2,3-cd]pyrene	2.09E-04	114.6954023	971.76
LAMININ BOSS	3.16E-04	27.82380506	224.23
LY-294002 PC3 UP	4.43E-04	77.29844961	596.93
aspirin	4.90E-04	13.95260323	106.34
Aflodac	5.23E-04	23.32349469	176.22
Benz[a]anthracene	1.04E-03	49.54975124	340.37
lamivudine	1.19E-03	46.09722222	310.25
Benzo[k]fluoranthene	0.0011941.19E-03	46.09722222	310.25

### 3.9 TIMER analysis

**FIGURE 13:**
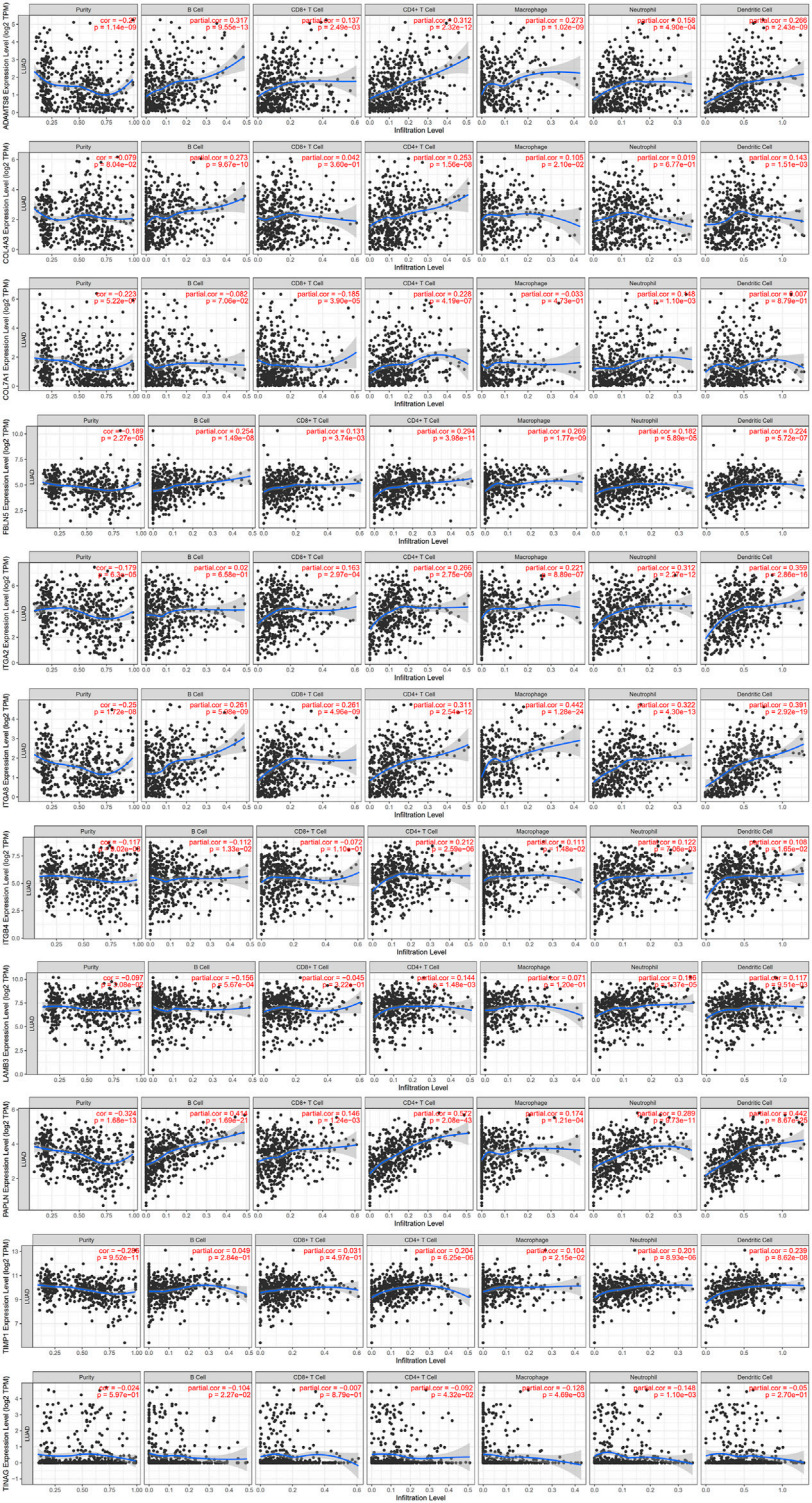
Immune cells infiltration by TIMER database.

The relationship between immune cells and 14 prognostic BM-related genes is crucial and was explored in this study based on the TIMER database. ADAMTS6, ADAMTS8, COL4A3, FBLN5, ITGA2, ITGA8, PAPLN, and TINAG showed positively correlation with immune cells, such as CD4+ T cells, B cells, CD8+ T cells, macrophages, dendritic cells, and neutrophils, whereas TINAG had negative correlation to these immune cells. COL7A1, ITGB4, and LAMB3 were negatively correlated with B cells and CD8+ T cells but had positive associations with CD4+ T cells, neutrophils, and dendritic cells, while COL7A1 was negatively associated with macrophages (Figure 13).

### 3.10 Drug sensitivity analysis

**FIGURE 14:**
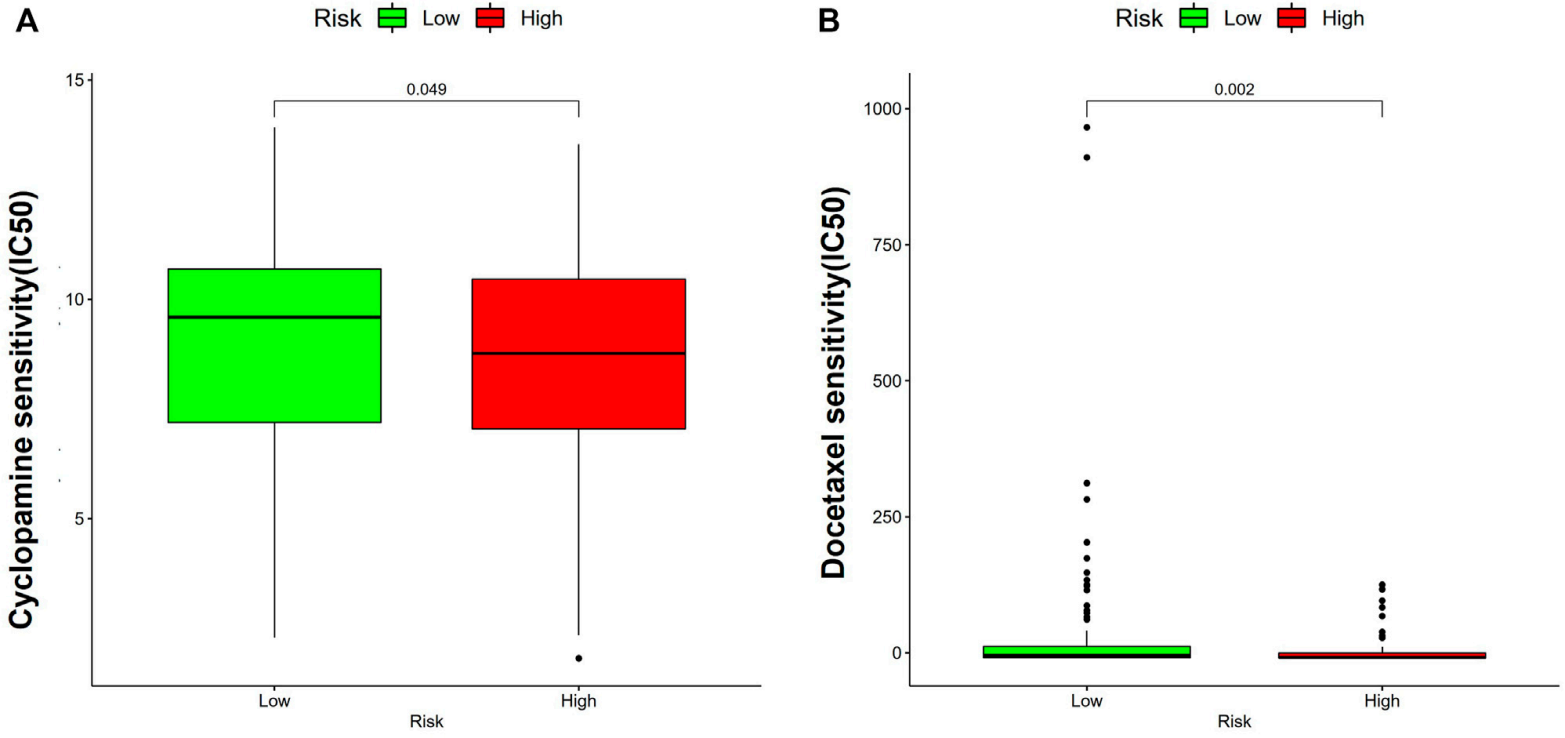
Drug sensitivity analysis. **(A)** Cyclopamine; **(B)** Docetaxel.

The study conducted a further investigated the differences in sensitivity to commonly used chemotherapy agents between high-risk and low-risk groups, to improve treatment effects. According to the GDSC database analysis, patients in the later group (high-risk) showed lower IC50 values of Cyclopamine and Docetaxel than those in the former group (low-risk), suggesting patients in the former group were more sensitive to the drugs (Figure 14).

## 4 Discussion

NSCLC accounts for approximately 85% of all LC cases worldwide. Most of them cannot be cured because of their complex nature and slow progression. Despite advances in molecular targeted therapy and immunotherapy, drug-based chemotherapy may modestly prolong the survival of these patients. However, current treatment outcomes appear to be stagnating, with little significant improvement in response rates or median survival status (Schiller et al., 2002; Scagliotti et al., 2008).

Many studies in recent years have focused on the importance of BM proteins, which are primarily involved in tumorigenesis and evolution. The pore size of transfer membranes may determine BM’s stiffness of the BM, which is dependent on the ratio of netrin-4 (NET-4) to laminin molecules. A larger ratio indicates softer BM, thus downregulating the invasive activity of cancer cells (Reuten et al., 2021). Meanwhile, its epithelial-mesenchymal transition enhances epithelial phenotype cells to be transformed into mesenchymal phenotype cells and metastasized through lymphatic vasculature via the metastatic cascade of intravenous and exosmosis, assisting cancer cells in spreading to remote organs from the tumor location (Banyard and Bielenberg, 2015). BM mainly consists of laminin, collagen, and integrins, all of which contribute to the metastasis of tumor cells, making them ideal targets for anticancer drugs (Xiao et al., 2015a; Rousselle and Scoazec, 2020; Su et al., 2020). Some recent studies have discovered that BM-related genes play a variety of functions during tumorigenesis; however, few have analyzed BM-related genes and comprehensively explored the clinical significance of BM in LUAD.

This study acquired 93 BM-related genes with different expressions in LUAD tissues and normal lung ones from TCGA database, and then conducted COX and LASSO regression analyses to assess the prognostic value of these BM-related genes and identified 14 genes associated with LUAD prognosis: TENM3, ADAMTS8, ADAMTS6, TINAG, ITGB4, LAMB3, TIMP1, ITGA8, COL7A1, FBLN5, COL4A3, PAPLN, SPARCL1, and ITGA2. Subsequently, a risk model which had association with the outcome was established and validated according to the 14 BM related genes. The survival and ROC analyses indicated a good predictive ability of this risk model. Results of AUCs with 1-, 3- and 5-year survival were 0.676, 0.687, and 0.703, respectively. Cox analysis determined that risk scores based on 14 BM-related genes could be an independent prognostic factor for LUAD. In addition, it was found that this signature was related to immune cell infiltration. Simultaneously, 12 small-molecule drugs have been discovered to treat LUAD.

The GO and KEGG analyses were conducted through the “limma” R package, aiming to probe into the potential mechanism of these BM genes in the risk model. Based on the GO enrichment analysis, differential expression was mainly connected with some process terms, namely, extracellular matrix organization and structure organization, together with external encapsulating structure organization. In accordance with KEGG enrichment analysis, BM related genes were involved in ECM− interactions and the PI3K−Akt signaling pathway. It should be mentioned that ECM is identified as one of the major components in the tumor microenvironment. It has been found Collagen is closely associated with ECM function, which may affect the biological behaviors of tumor cells (Riegler et al., 2018). Epithelial functions, including cell differentiation, migration, and invasion, are mediated by physical interactions with the ECM (Lu et al., 2012). Su et al. (2021) used a bioinformatics method to use microarray data and enriched differentially expressed genes in lung cancer via the ECM-receptor interaction pathway. This is consistent with our results, which indicate that this pathway is crucial for the development and occurrence of LUAD. The PI3K-AKT pathway, a classic cancer-related signaling pathway, increases endothelial tube formation and survival when activated (Cheng et al., 2017). These pathways are closely related to the malignant phenotype of many cancers, suggesting that BM-related genes play key roles in tumorigenesis and development. In addition, 14 BM-related genes of prognostic value were associated with immune cell infiltration, according to TIMER database analysis, suggesting that BM-related genes may promote cancer progression by regulating immune infiltration.

This study identified 14 BM-related genes: TENM3, ADAMTS8, ADAMTS6, TINAG, ITGB4, LAMB3, TIMP1, ITGA8, COL7A1, FBLN5, COL4A3, PAPLN, SPARCL1, and ITGA2), and concluded that these genes may be capable of predicting the OS of patients with LUAD.

TENM3, a member of the conserved Teneurin family, is a highly conserved transmembrane glycoprotein receptor associated with tumor development and drug resistance (Ziegler et al., 2012). The relationship between TENM3 and neuroblastoma has been reported by Hiwatari et al. (Hiwatari et al., 2022), who suggested that TENM3 acts as a novel ALK partner in young adults at high risk for stage 4 neuroblastoma. However, few research has been conducted on this gene in LC, the only studies found that the mutation frequency of TENM3 increased significantly in patients with stage III (Liu et al., 2023). Our results can provide clues for future basic and clinical research related to LUAD treatment.

ADAMTS8 serves as a suppressor or oncogene in numerous cancers (Zhong and Khalil, 2019). In breast cancer, its overexpression may be associated with longer OS and progression-free survival, which may take part in cell cycle regulation, and may be related to the EGFR/Akt signaling pathway (Zhang et al., 2022a). Based on some findings, it was lower in NSCLC tissues compared with normal tissues (Dunn et al., 2004). Zhang et al. (2022b) reported that high ADAMTS8 levels had association with higher survival in LC patients. Moreover, it was abnormally downregulated in NSCLC cells. The upregulated proteins may inhibit cell proliferation and promote apoptosis by suppressing VEGFA. Lee et al. (2022) found that ADAMTS8 expression was positively correlated with the recruitment of anti-cancer NKT cells, patients with higher ADAMTS8 levels in wild-type EGFR or low PD-L1 groups survived longer than with low levels of LUAD, suggesting that ADAMTS8 may be a treatment option for patients with lung adenoma who lack effective targeted or immunotherapy. ADAMTS8 acts as a secretory protease to inhibit the EGFR signaling pathway, while phosphorylating MEK and ERK levels are reduced. ADAMTS8 also acts as a functional tumor suppressor by antagonizing EGFR-MEK-ERK signaling to disrupt actin stressed fibrous tissue and inhibit tumor cell motility, which is often methylated in common tumors (Choi et al., 2014).

ADAMTS6 is one of members in the ADAMTS family, playing a key role in regulating the progression of many cancers spanning several organ systems, such as breast cancer (Xu et al., 2021) and colorectal cancer (Xiao et al., 2015b). A study using TCGA and RNA-seq data showed that its high expression may be related to poor clinical outcomes in patients with gastric cancer (Zhu et al., 2021). In our analysis, ADAMTS6 was highly expressed in tissues of LUAD patients, mainly in high-risk patients. Luu et al. (2020) found that ADAMTS6 is associated with NSCLC by modulating AGR2, a recently discovered oncogene related to p53. The relationship between ADAMTS6 and NSCLC was further described by Lachat et al. (2020) chromatin immunoprecipitation and qRT-PCR analysis showed that ADAMTS6 expression was upregulated more than 60-fold during EMT and was associated with deletion of the promoter H3K27me3.

TINAG encodes extracellular matrix proteins that are expressed in the tubular BM. Through a literature search, we found that TINAG plays a major role in regulating kidney-related diseases; however, there are few studies on cancer. Zhang et al. (2018) reported TINAG was highly expressed in hepatocellular carcinoma, which was related to three factors: pathological metastasis, pathological stage, and pathological node. It has also been confirmed that PI3K/AKT activation plays a major role in promoting TINAG-mediated migration, proliferation, and invasion. TINAG is a key gene related to the prognosis of pancreatic cancer (Liu et al., 2019). Our results showed that TINAG is highly expressed in LUAD, which is the first time that this gene has been reported in LUAD. Further studies and verifications are required.

Integrins regulate a variety of cellular behaviors in response to cytokines and growth factors by forming transmembrane connections between the extracellular matrix (ECM) and actin cytoskeleton (Thuveson et al., 2019). ITGB4 was upregulated in various tumor types (Mercurio et al., 2001; Thuveson et al., 2019) and was linked to poor prognosis or aggressive behavior (Kurokawa et al., 2008). Mohanty et al. (2020) recently reported that high ITGB4 expression has association with poor prognosis in LUAD patients, which is consistent with our results. Ning et al. (2022) found that ZBM-H, a HOCl probe, inhibited the migration of A549 cells by inducing autophagy to negatively regulate ITGB4 protein levels, suggesting that ITGB4 might act as a diagnostic biomarker of LUAD. As a transmembrane receptor for collagen and related proteins, ITGA2 has been shown to influence cancer progression through multiple pathways (Ghosh et al., 2016). The upregulation and relocalization of ITGA2 may promote tumor metastasis by increasing adhesion to the ECM and inhibiting local adhesion kinase activation. Studies have demonstrated that transient ITGA2 knockdown in NSCLC inhibits TNBC proliferation by inducing a G1 block (Adorno-Cruz et al., 2020). Chen et al. (2021) demonstrated that long non-coding RNA SLC25A25-AS1 plays a carcinogenic role in NSCLC by regulating the miRNA-195-5p/ITGA2 axis, suggesting the involvement of ITGA2 in the progression of NSCLC. To date, several studies have linked ITGA8 to tumorigenesis (Matsushima et al., 2020), and Lu et al. (2016) used bioinformatics methods to demonstrate that low ITGA8 expression had association with poor disease-free prognosis and survival in clear cell RCC patients. Similarly, through multivariate Cox regression analysis, Cui et al. (2022) discovered that ITGA8 had connection with poor prognosis in NSCLC and could considered as an independent predictor. Li et al. (2023) found that miR-17-5p could regulate the expression of ITGA8. Therefore, the regulatory network of ITGA8 may be a new therapeutic target to improve the prognosis of LC patients.

Studies have found that LAMB3 may participate in invasion and metastasis ability of some cancers, like thyroid, liver, and lung cancers (Wang et al., 2013; Wang et al., 2017; Hou et al., 2018). Zhang et al. (2019) verified that LAMB3 mediates apoptosis, proliferation, invasion, and metastasis of pancreatic cancers through the regulation of the PI3K/Akt signaling pathway. As a key gene involved in the progression of lung cancer, it showed high expression in LC tissues compared with normal ones (Wang et al., 2013).

Type VII collagen is encoded by COL7A1. Any changes in collagen under the tumor microenvironment will be reflected in the aspects of new collagen, density, direction, length, and cross-linking, which may influence many aspects through the regulation of epithelial-mesenchymal transformation, immunity, and mesenchymal cells, namely, tumor cell metabolism, macromolecular transport, gene expression, angiogenesis, as well as tumor invasion and metastasis (Peng et al., 2016). It was confirmed in this study that COL7A1 served as an oncogene in LUAD because its expression was upregulated compared to that in normal tissues. Li et al. (2020) claimed that COL7A1 may act as a crucial gene affecting the progression of hypoxia-related LUAD via DNA methylation. Unlike COL7A1, COL4A3 is expressed at low levels in lung cancer tissue. Metodieva et al. (2011) found that the expression of COL4A3 genes was upregulated in early NSCLC, while in another study, patients with lower COL4A3 expression shared a longer median OS (Jiang et al., 2013), confirming that it may act as a biomarker of LC. It has been reported that DNA repair can be weakened by blocking the interaction between COL4A3 proteins (Stabuc-Silih et al., 2009).

FBLN5, as a member of the fibulin family, is of great importance in angiogenesis and elastic fiber assembly. FBLN5 is often silenced by promoter hypermethylation in NSCLC, which is the main reason why it is often downregulated in more than 50% of lung cancers (Yue et al., 2009). FBLN5 may slow down the metastasis and invasion of lung cancer by inhibiting MMP-7 expression and promoting tumor metastasis through BM degradation. At the same time, it may also act as a barrier for surrounding tissues (Yue et al., 2009; Chen et al., 2014), and Chen et al. (2015) demonstrated that this process may be conducted by regulating the Wnt/β-catenin pathway. Little research has been conducted on the link between PAPLN and cancer. In studying cell-cell crosstalk in extramedullary infiltration of acute myeloid leukemia, Lv et al. (2018) found that PALPN showed a protective effect against the disease, thereby prolonging the overall survival. It was found that SPARCL1 is downregulated in many histological types of human epithelial cancers, including NSCLC (Sullivan and Sage, 2004). The role of SPARCL1 in NSCLC biology has been widely discussed. By cloning and mapping human SPARCL1 genomic loci and using FISH technology, Isler et al. (2001) found that SPARCL1 acts as a tumor suppressor. Zhou and Zhang (2021) found that differences in SPARCL1 expression in NSCLC was connected with clinical stage, and the low expression rate of SPARCL1 in the TNM stage (I-II) was higher than that in the TNM stage (III-IV). Patients with higher SPARCL1 expression had higher 5-year survival rates. The above evidence indicated FBLN5 plays a major role in the prognosis and development of NSCLC. However, it has been previously reported that SPARCL1 activates typical WNT/β-catenin signaling by stabilizing the Wnt-receptor complex, inhibiting osteosarcoma metastasis and recruiting macrophages (Zhao et al., 2018), but the signaling pathway through which SPARCL1 is involved in the LUAD process remains unknown.

In accordance with the GSEA analysis, BM-related genes were mainly involved in metabolism-related pathways, including the Fc epsilon RI signaling pathway, which also plays a role in linoleic acid metabolism, Phagosome, *Salmonella* infection, and spliceosome, with main enrichment in the high-risk group. Therefore, BM-based markers were demonstrated to be able to predict the prognosis of LUAD patients and might have a significant function in LUAD treatment.

CD4^+^T cells indicated an excellent prognosis and outstanding response to pembrolizumab, which had same results in this study. That is, patients in the former group had less CD4^+^ T cells, while those in the latter group had higher expression of CD276, HHLA2, and TNFSF4, indicating that poor prognosis may be related to the immunosuppressive microenvironment. Besides, patients in low-risk populations may benefit from checkpoint inhibitor immunotherapy. Additionally, according to our findings, LUAD suffers in the low-risk group may profit by Cyclopamine and Docetaxel.

In this study, 14 BM-related genes involved in LUAD process were found, and the variation of these genes is the basis of human diseases. The BM protein is the target of autoantibodies in immune diseases. BM proteins are targets of autoantibodies in immune disorders and defects in BM protein expression and turnover are a key pathogenic aspect of cancer. Although we used bioinformatics to identify prognostic BM-related genes involved in LUAD, the limitations in our study should be seen. The proposed verification cohort was based on the retrospective TCGA data. Therefore, additional validation of the model should be performed in massive-sample clinical research. We expect that this will ultimately facilitate early disease detection, improve prognostic prediction, and inform the treatment of BM-associated cancers, including LUAD.

## 5 Conclusion

Overall, this study identified differentially expressed BM-related genes, which may be involved in cancer, together with progression of NSCLC. These genes are valuable to predict the prognosis of NSCLC patients. At the same time, targeting BM-related genes is expected to be an effective treatment for NSCLC. It shall be mentioned that this study may be further confirmed by other research in the future.

## Data Availability

The original contributions presented in the study are included in the article/Supplementary Material, further inquiries can be directed to the corresponding author.

## Abbreviations

LUAD, lung adenocarcinoma; bm, basement membrane; TCGA, The Cancer Genome Atlas; LC, lung cancer; NSCLC, non-small cell lung cancer; LASSO, The least absolute shrinkage and selection operator; GO, Gene Ontology; CC, Cell Composition; MF, Molecular Functionality; BP, Biological Process; KEGG, Kyoto Encyclopedia of Genes and Genomes; STRING, the Search Tool for the Retrieval of Distant Genes Database; CMAP, Connectivity MAP database; ROC, receiver operating characteristic; GSEA, Gene Set Enrichment Analysis; GDSC, Genomics of Drug Sensitivity in Cancer; EMT, epithelial-Mesenchymal transition.

## References

[B1] Adorno-Cruz V. Hoffmann A. D. Liu X. Dashzeveg N. K. Taftaf R. Wray B. (2020). ITGA2 promotes expression of ACLY and CCND1 in enhancing breast cancer stemness and metastasis. Genes Dis. 8 (4), 493–508. 10.1016/j.gendis.2020.01.015 34179312 PMC8209312

[B2] Banyard J. Bielenberg D. R. (2015). The role of EMT and MET in cancer dissemination. Connect. Tissue Res. 56 (5), 403–413. 10.3109/03008207.2015.1060970 26291767 PMC4780319

[B3] Celentano A. Yap T. Paolini R. Yiannis C. Mirams M. Koo K. (2021). Inhibition of matrix metalloproteinase-2 modulates malignant behaviour of oral squamous cell carcinoma cells. J. Oral Pathol. Med. 50 (3), 323–332. 10.1111/jop.12992 31925966

[B4] Chen J. Gao C. Zhu W. (2021). Long non-coding RNA SLC25A25-AS1 exhibits oncogenic roles in non-small cell lung cancer by regulating the microRNA-195-5p/ITGA2 axis. Oncol. Lett. 22 (1), 529. 10.3892/ol.2021.12790 34055094 PMC8138898

[B5] Chen X. Meng J. Yue W. Yu J. Yang J. Yao Z. (2014). Fibulin-3 suppresses Wnt/β-catenin signaling and lung cancer invasion. Carcinogenesis 35 (8), 1707–1716. 10.1093/carcin/bgu023 24480807 PMC4123641

[B6] Chen X. Song X. Yue W. Chen D. Yu J. Yao Z. (2015). Fibulin-5 inhibits Wnt/β-catenin signaling in lung cancer. Oncotarget 6 (17), 15022–15034. 10.18632/oncotarget.3609 25909283 PMC4558133

[B7] Cheng H.-W. Chen Y.-F. Wong J.-M. Weng C.-W. Chen H.-Y. Yu S.-L. (2017). Cancer cells increase endothelial cell tube formation and survival by activating the PI3K/Akt signalling pathway. J. Exp. Clin. Cancer Res. 36 (1), 27. 10.1186/s13046-017-0495-3 28173828 PMC5296960

[B8] Choi G.-C. Li J. Wang Y. Li L. Zhong L. Ma B. (2014). The metalloprotease ADAMTS8 displays antitumor properties through antagonizing EGFR-MEK-ERK signaling and is silenced in carcinomas by CpG methylation. Mol. Cancer Res. 12 (2), 228–238. 10.1158/1541-7786.MCR-13-0195 24184540

[B9] Cui S. Lou S. Feng J. Tang X. Xiao X. Huang R. (2022). Identification of genes and pathways leading to poor prognosis of non-small cell lung cancer using integrated bioinformatics analysis. Transl. Cancer Res. 11 (4), 710–724. 10.21037/tcr-21-1986 35571642 PMC9091047

[B10] Danilova L. Ho W.-J. Zhu Q. Vithayathil T. DeJesus-Acosta. A. Azad N. S. (2019). Programmed cell death ligand-1 (PD-L1) and CD8 expression profiling identify an immunologic subtype of pancreatic ductal adenocarcinomas with favorable survival. Cancer Immunol. Res. 7 (6), 886–895. 10.1158/2326-6066.CIR-18-0822 31043417 PMC6548624

[B11] Duma N. Santana-Davila R. Molina J. R. (2019). Non-small cell lung cancer: Epidemiology, screening, diagnosis, and treatment. Mayo Clin. Proc. 94 (8), 1623–1640. 10.1016/j.mayocp.2019.01.013 31378236

[B12] Dunn J. R. Panutsopulos D. Shaw M. W. Heighway J. Dormer R. Salmoal. E. N. (2004). METH-2 silencing and promoter hypermethylation in NSCLC. Br. J. Cancer 13 (6), 1149–1154. 10.1038/sj.bjc.6602107 PMC2747718 15328519

[B13] Ghosh S. Shinogle H. E. Galeva N. A. Dobrowsky R. T. Blagg B. S. (2016). Endoplasmic reticulum-resident heat shock protein 90 (HSP90) isoform glucose-regulated protein 94 (GRP94) regulates cell polarity and cancer cell migration by affecting intracellular transport. J. Biol. Chem. 291 (16), 8309–8323. 10.1074/jbc.M115.688374 26872972 PMC4861406

[B14] Götte M. Kovalszky I. (2018). Extracellular matrix functions in lung cancer. Matrix Biol. 73, 105–121. 10.1016/j.matbio.2018.02.018 29499357

[B15] Herbst R. S. Morgensztern D. Boshoff C. (2018). The biology and management of non-small cell lung cancer. Nature 553 (7689), 446–454. 10.1038/nature25183 29364287

[B16] Hiwatari M. Seki M. Matsuno R. Yoshida K. Nagasawa T. Sato-Otsubo A. (2022). Novel TENM3-ALK fusion is an alternate mechanism for ALK activation in neuroblastoma. Oncogene 41 (20), 2789–2797. 10.1038/s41388-022-02301-1 35411036

[B17] Hou J. Wang L. Wu D. (2018). The root of Actinidia chinensis inhibits hepatocellular carcinomas cells through LAMB3. Cell Biol. Toxicol. 34 (4), 321–332. 10.1007/s10565-017-9416-7 29127567

[B18] Isler S. G. Schenk S. Bendik I. Schraml P. Novotna H. Moch H. (2001). Genomic organization and chromosomal mapping of SPARC-like 1, a gene down regulated in cancers. Int. J. Oncol. 18 (3), 521–526. 10.3892/ijo.18.3.521 11179481

[B19] Januchowski R. Świerczewska M. Sterzyńska K. Wojtowicz K. Nowicki M. Zabel M. (2016). Increased expression of several collagen genes is associated with drug resistance in ovarian cancer cell lines. J. Cancer 7 (10), 1295–1310. 10.7150/jca.15371 27390605 PMC4934038

[B20] Jayadev R. Morais M. R. P. T. Ellingford J. M. Srinivasan S. Naylor R. W. Lawless C. (2022). A basement membrane discovery pipeline uncovers network complexity, regulators, and human disease associations. Sci. Adv. 20 (20), eabn2265. 10.1126/sciadv.abn2265 PMC9116610 35584218

[B21] Jiang C.-P. Wu B.-H. Chen S.-P. Fu M.-Y. Yang M. Liu F. (2013). High COL4A3 expression correlates with poor prognosis after cisplatin plus gemcitabine chemotherapy in non-small cell lung cancer. Tumour Biol. 34 (1), 415–420. 10.1007/s13277-012-0565-2 23108892

[B22] Kurokawa A. Nagata M. Kitamura N. Noman A. A. Ohnishi M. Ohyama T. (2008). Diagnostic value of integrin alpha3, beta4, and beta5 gene expression levels for the clinical outcome of tongue squamous cell carcinoma. Cancer 112 (6), 1272–1281. 10.1002/cncr.23295 18224668

[B23] Lachat C. Bruyère D. Etcheverry A. Aubry M. Mosser J. Warda W. (2020). EZH2 and KDM6B expressions are associated with specific epigenetic signatures during EMT in non small cell lung carcinomas. Cancers (Basel) 12 (12), 3649. 10.3390/cancers12123649 33291363 PMC7762040

[B24] Lee H.-C. Chang C.-Y. Wu K.-L. Chiang H.-H. Chang Y.-Y. Liu L.-X. (2022). The therapeutic potential of ADAMTS8 in lung adenocarcinoma without targetable therapy. J. Pers. Med. 12 (6), 902. 10.3390/jpm12060902 35743687 PMC9225423

[B25] Li H. Tong L. Tao H. Liu Z. (2020). Genome-wide analysis of the hypoxia-related DNA methylation-driven genes in lung adenocarcinoma progression. Biosci. Rep. 40 (2), BSR20194200. 10.1042/BSR20194200 32031203 PMC7033312

[B26] Li X. Zhu G. Li Y. Huang H. Chen C. Wu D. (2023). LINC01798/miR-17-5p axis regulates ITGA8 and causes changes in tumor microenvironment and stemness in lung adenocarcinoma. Front. Immunol. 23 (14), 1096818. 10.3389/fimmu.2023.1096818 PMC9995370 36911684

[B27] Liu C.-C. Lin J.-H. Hsu T.-W. Hsu J.-W. Chang J.-W. Su K. (2016). Collagen XVII/laminin-5 activates epithelial-to-mesenchymal transition and is associated with poor prognosis in lung cancer. Oncotarget 9 (2), 1656–1672. 10.18632/oncotarget.11208 29416721 PMC5788589

[B28] Liu J. Shen J.-X. Wu H.-T. Li X.-L. Wen X.-F. Du C.-W. (2018). Collagen 1A1 (COL1A1) promotes metastasis of breast cancer and is a potential therapeutic target. Discov. Med. 25 (139), 211–223. 29906404

[B29] Liu Y. Duan J. Zhang F. Liu F. Luo X. Shi Y. (2023). Mutational and transcriptional characterization establishes prognostic models for resectable lung squamous cell carcinoma. Cancer Manag. Res. 17 (15), 147–163. 10.2147/CMAR.S384918 PMC9942504 36824152

[B30] Liu Y. Zhu D. Xing H. Hou Y. Sun Y. (2019). A 6-gene risk score system constructed for predicting the clinical prognosis of pancreatic adenocarcinoma patients. Oncol. Rep. 41 (3), 1521–1530. 10.3892/or.2019.6979 30747226 PMC6365694

[B31] Lu P. Weaver V. M. Werb Z. (2012). The extracellular matrix: A dynamic niche in cancer progression. J. Cell Biol. 196 (4), 395–406. 10.1083/jcb.201102147 22351925 PMC3283993

[B32] Lu X. Wan F. Zhang H. Shi G. Ye D. (2016). ITGA2B and ITGA8 are predictive of prognosis in clear cell renal cell carcinoma patients. Tumour Biol. 37 (1), 253–262. 10.1007/s13277-015-3792-5 26198048

[B33] Luu T.-T. Bach D. H. Kim D. Hu R. Park H. J. Lee S. K. (2020). Overexpression of AGR2 is associated with drug resistance in mutant non-small cell lung cancers. Anticancer Res. 40 (4), 1855–1866. 10.21873/anticanres.14139 32234873

[B34] Lv C. Sun L. Guo Z. Li H. Kong D. Xu B. (2018). Circular RNA regulatory network reveals cell-cell crosstalk in acute myeloid leukemia extramedullary infiltration. J. Transl. Med. 16 (1), 361. 10.1186/s12967-018-1726-x 30558617 PMC6297994

[B35] Matsushima S. Aoshima Y. Akamatsu T. Enomoto Y. Meguro S. Kosugi. (2020). CD248 and integrin alpha-8 are candidate markers for differentiating lung fibroblast subtypes. BMC Pulm. Med. 21 (1), 21. 10.1186/s12890-020-1054-9 PMC6975017 31964365

[B36] Mercurio A. M. Bachelder R. E. Rabinovitz I. O'Connor K. L. Tani T. Shaw L.-M. (2001). The metastatic odyssey: The integrin connection. Surg. Oncol. Clin. N. Am. 10 (2), 313–328. 10.1016/s1055-3207(18)30067-x 11382589

[B37] Metodieva S. N. Nikolova D. N. Cherneva R. V. Dimova I. I. Petrov D. B. Toncheva D. I. (2011). Expression analysis of angiogenesis-related genes in Bulgarian patients with early-stage non-small cell lung cancer. Tumor 97 (1), 86–94. 10.1177/030089161109700116 21528670

[B38] Mohanty A. Nam A. Pozhitkov A. Yang L. Srivastava S. (2020). A non-genetic mechanism involving the integrin β4/paxillin Axis contributes to chemoresistance in lung cancer. Science 23 (9), 101496. 10.1016/j.isci.2020.101496 PMC7502350 32947124

[B39] Ning J. Cui X. Li N. Li N. Zhao B. Miao J. (2022). Activation of GRP78 ATPase suppresses A549 lung cancer cell migration by promoting ITGB4 degradation. Cell Adh Migr. 16 (1), 107–114. 10.1080/19336918.2022.2130415 36203272 PMC9542429

[B40] Peng D.-H. Ungewiss C. Tong P. Byers L. A. Wang J. Canales J. R. (2016). ZEB1 induces LOXL2-mediated collagen stabilization and deposition in the extracellular matrix to drive lung cancer invasion and metastasis. Oncogene 36 (14), 1925–1938. 10.1038/onc.2016.358 27694892 PMC5378666

[B41] Reuten R. Zendehroud S. Nicolau M. Fleischhauer L. Laitala A. Kiderlen S. (2021). Basement membrane stiffness determines metastases formation. Nat. Mater 20 (6), 892–903. 10.1038/s41563-020-00894-0 33495631

[B42] Riegler J. Labyed Y. Rosenzweig S. Javinal V. Castiglioni A. (2018). Tumor elastography and its association with collagen and the tumor microenvironment. Clin. Cancer Res. 24 (18), 4455–4467. 10.1158/1078-0432.CCR-17-3262 29798909

[B43] Rousselle P. Scoazec J. Y. (2020). Laminin 332 in cancer: When the extracellular matrix turns signals from cell anchorage to cell movement. Semin. Cancer Biol. 62, 149–165. 10.1016/j.semcancer.2019.09.026 31639412

[B44] Scagliotti G. V. Parikh P. von Pawel J. Biesma B. Vansteenkiste J. Manegold C. (2008). Phase III study comparing cisplatin plus gemcitabine with cisplatin plus pemetrexed in chemotherapy-naive patients with advanced-stage non-small-cell lung cancer. J. Clin. Oncol. 26 (21), 3543–3551. 10.1200/JCO.2007.15.0375 18506025

[B45] Schiller J. H. Harrington D. Belani C. P. Langer C. Sandler A. Krook J. (2002). Comparison of four chemotherapy regimens for advanced non-small-cell lung cancer. N. Engl. J. Med. 346 (2), 92–98. 10.1056/NEJMoa011954 11784875

[B46] Siegel R. L. Miller K. D. Jemal A. (2020). Cancer statistics, 2020. CA Cancer J. Clin. 70 (1), 7–30. 10.3322/caac.21590 31912902

[B47] Stabuc-Silih M. Ravnik-Glavac M. Glavac D. Hawlina M. Strazisar M. (2009). Polymorphisms in COL4A3 and COL4A4 genes associated with keratoconus. Mol. Vis. 15, 2848–2860. 20029656 PMC2796875

[B48] Su C.-Y. Li J.-Q. Zhang L.-L. Wang H. Wang F.-H. Tao Y.-W. (2020). The biological functions and clinical applications of integrins in cancers. Front. Pharmacol. 11, 57906. 10.3389/fphar.2020.579068 PMC7522798 33041823

[B49] Su C. Liu W.-X. Wu L.-S. Dong T.-J. Liu J.-F. (2021). Screening of hub gene targets for lung cancer via microarray data. Comb. Chem. High. Throughput Screen 24 (2), 269–285. 10.2174/1386207323666200808172631 32772911

[B50] Sullivan M. M. Sage E. H. (2004). Hevin/SC1, a matricellular glycoprotein and potential tumor-suppressor of the SPARC/BM-40/Osteonectin family. Int. J. Biochem. Cell Biol. 36 (6), 991–996. 10.1016/j.biocel.2004.01.017 15094114

[B51] Tas F. Bilgin E. Tastekin D. Erturk K. Duranyildiz D. (2016). Serum IGF-1 and IGFBP-3 levels as clinical markers for patients with lung cancer. Biomed. Rep. 4 (5), 609–614. 10.3892/br.2016.629 27123256 PMC4840770

[B52] Teng Y. Wang Z. Ma L. Zhang L. Guo Y. Gu M. (2016). Prognostic significance of circulating laminin gamma2 for early-stage non-small-cell lung cancer. Onco Targets Ther. 7 (9), 4151–4162. 10.2147/OTT.S105732 PMC4939988 27462170

[B53] Thuveson M. Gaengel K. Collu G. M. Chin M. L. Singh J. Mlodzik M. (2019). Integrins are required for synchronous ommatidial rotation in the Drosophila eye linking planar cell polarity signalling to the extracellular matrix. Open Biol. 9 (8), 190148. 10.1098/rsob.190148 31409231 PMC6731590

[B54] Wang X.-M. Li J. Yan M.-X. Liu L. Jia D.-S. Geng Q. (2013). Integrative analyses identify osteopontin, LAMB3 and ITGB1 as critical pro-metastatic genes for lung cancer. PLoS One 8 (2), e55714. 10.1371/journal.pone.0055714 23441154 PMC3575388

[B55] Wang Y. Jin Y. Bhandari A. Yao Z. Yang F. Pan Y. (2017). Upregulated LAMB3 increases proliferation and metastasis in thyroid cancer. Onco Targets Ther. 21 (11), 37–46. 10.2147/OTT.S149613 PMC5743181 29317832

[B56] Xiao Q. Jiang Y. Liu Q. Yue J. Liu C. Zhao X. (2015a). Minor type IV collagen α5 chain promotes cancer progression through discoidin domain receptor-1. PLoS Genet. 11 (5), e1005249. 10.1371/journal.pgen.1005249 25992553 PMC4438069

[B57] Xiao Q. Qu X.-L. Li X.-M. Sun Y.-L. Zhao H.-X. Wang S. (2015b). Identification of commonly dysregulated genes in colorectal cancer by integrating analysis of RNA-Seq data and qRT-PCR validation. Cancer Gene Ther. 22 (5), 278–284. 10.1038/cgt.2015.20 25908452

[B58] Xu X. Li N. Wang Y. Yu J. Mi J. (2021). Calcium channel TRPV6 promotes breast cancer metastasis by NFATC2IP. Cancer Lett. 28 (519), 150–160. 10.1016/j.canlet.2021.07.017 34265397

[B59] Yue W. Sun Q. Landreneau R. Wu C. Siegfried J. M. Yu J. (2009). Fibulin-5 suppresses lung cancer invasion by inhibiting matrix metalloproteinase-7 expression. Cancer Res. 69 (15), 6339–6346. 10.1158/0008-5472.CAN-09-0398 19584278 PMC2719681

[B60] Yurchenco P. D. (2011). Basement membranes: Cell scaffoldings and signaling platforms. Cold Spring Harb. Perspect. Biol. 3 (2), a004911. 10.1101/cshperspect.a004911 21421915 PMC3039528

[B61] Zhang H. Pan Y.-Z. Cheung M. Cao M. Yu C. Chen L. (2019). LAMB3 mediates apoptotic, proliferative, invasive, and metastatic behaviors in pancreatic cancer by regulating the PI3K/Akt signaling pathway. Cell Death Dis. 10 (3), 230. 10.1038/s41419-019-1320-z 30850586 PMC6408539

[B62] Zhang M.-H. Niu H. Li Z. Huo R.-T. Wang J.-M. Liu J. (2018). Activation of PI3K/AKT is involved in TINAG-mediated promotion of proliferation, invasion and migration of hepatocellular carcinoma. Cancer Biomark. 23 (1), 33–43. 10.3233/CBM-181277 29991125 PMC13078558

[B63] Zhang Q. Hu K. Qu Z. Xie Z. Tian F. (2022b). ADAMTS8 inhibited lung cancer progression through suppressing VEGFA. Biochem. Biophys. Res. Commun. 2 (598), 1–8. 10.1016/j.bbrc.2022.01.110 35149432

[B64] Zhang Q. Kanyomse Q. Luo C. Mo Q. Zhao X. Wang L. (2022a). The prognostic value of ADAMTS8 and its role as a tumor suppressor in breast cancer. Cancer Invest. 11, 119–132. 10.1080/07357907.2022.2128367 36346393

[B65] Zhao S.-J. Jiang Y.-Q. Xu N.-W. Li Q. Zhang Q. Wang S.-Y. (2018). SPARCL1 suppresses osteosarcoma metastasis and recruits macrophages by activation of canonical WNT/β-catenin signaling through stabilization of the WNT-receptor complex. Oncogene 37 (8), 1049–1061. 10.1038/onc.2017.403 29084211 PMC5851113

[B66] Zhong S. Khalil R. A. (2019). A Disintegrin and Metalloproteinase (ADAM) and ADAM with thrombospondin motifs (ADAMTS) family in vascular biology and disease. Biochem. Pharmacol. 164, 188–204. 10.1016/j.bcp.2019.03.033 30905657 PMC6580420

[B67] Zhou Y. Zhang Q. (2021). Association of tumor suppressor Sparcl1 with clinical staging and prognosis of NSCLC. Ann. Clin. Lab. Sci. 51 (6), 756–765. 34921028

[B68] Zhu Y.-Z. Liu Y. Liao X.-W. Luo S.-S. (2021). Identified a disintegrin and metalloproteinase with thrombospondin motifs 6 serve as a novel gastric cancer prognostic biomarker by bioinformatics analysis. Biosci. Rep. 41 (4), BSR20204359. 10.1042/BSR20204359 33851708 PMC8065180

[B69] Ziegler A. Corvalán A. Roa I. Brañes J. A. Wollscheid B. (2012). Teneurin protein family: An emerging role in human tumorigenesis and drug resistance. Cancer Lett. 326 (1), 1–7. 10.1016/j.canlet.2012.07.021 22841666

